# First report of *Giardia duodenalis* and *Enterocytozoon bieneusi* in forest musk deer (*Moschus berezovskii*) in China

**DOI:** 10.1186/s13071-018-2681-3

**Published:** 2018-03-26

**Authors:** Yuan Song, Wei Li, Haifeng Liu, Zhijun Zhong, Yan Luo, Yao Wei, Wenlong Fu, Zhihua Ren, Ziyao Zhou, Lei Deng, Jianguo Cheng, Guangneng Peng

**Affiliations:** 1 The Key Laboratory of Animal Disease and Human Health of Sichuan Province, College of Veterinary Medicine, Sichuan Agricultural University, Chengdu, Sichuan Province China; 2 Sichuan Institute of Musk Deer Breeding, Dujiangyan, Sichuan Province China

**Keywords:** *G. duodenalis*, *E. bieneusi*, Zoonotic pathogens, Musk deer, China

## Abstract

**Background:**

*Giardia duodenalis* and *Enterocytozoon bieneusi* are widespread pathogens that can infect humans and various animal species. Thus far, there are only a few reports of *G. duodenalis* and *E. bieneusi* infections in ruminant wildlife. Thus, the objective of this study was to examine the prevalence of *G. duodenalis* and *E. bieneusi* in forest musk deer in Sichuan, China, as well as identifying their genotypes.

**Results:**

In total, we collected 223 faecal samples from musk deer at the Sichuan Institute of Musk Deer Breeding in Dujiangyan (*n* = 80) and the Maerkang Breeding Institute (*n* = 143). Five (2.24%) faecal samples were positive for *G. duodenalis*; three belonged to assemblage E, and two belonged to assemblage A based on the sequence analysis of the β-giardin (*bg*) gene. One sample each was found to be positive based on the glutamate dehydrogenase (*gdh*) and triosephosphate isomerase (*tpi*) gene, respectively. Thirty-eight (17.04%) faecal samples were found to be *E. bieneusi-*positive based on the internal transcribed spacer (ITS) sequence, and only SC03 genotype was identified, which belonged to the zoonotic group 1 according to the phylogenic analysis. The infection rates were significantly different among the different geographical areas and age groups but had no apparent association with gender or clinical symptoms.

**Conclusions:**

To our knowledge, this was the first molecular characterisation of *G. duodenalis* and *E. bieneusi* in musk deer. Identification of the zoonotic genotypes indicated a potential public health threat, and our study suggested that the forest musk deer is an important carrier of these parasites.

## Background

*Giardia* spp. are parasites with a broad host range comprising economic, companion, and wildlife animals, ranging from mammals to amphibians and birds, and humans [1, 2]. These parasites can have various clinical manifestations such as diarrhoea and abnormalities in growth and development, particularly in young hosts. For example, giardiasis can develop into malabsorption syndromes and other chronic diseases, resulting in stunted growth or emaciation in children [3]. According to WHO, approximately 200 million people in Africa, Asia and Latin America have symptomatic *Giardia* infection [4]. *Enterocytozoon bieneusi* is another common intestinal parasite that infects the host’s enterocytes, causing gastrointestinal illness such as chronic diarrhoea in animals and humans, particularly in immunosuppressed groups, including organ-transplant recipients, children, the elderly, and patients with cancer, diabetes, or AIDS [5, 6]. Ingestion of water and food contaminated with oocyst-containing faeces is the principal route of transmission for these species [7].

*Giardia duodenalis* is the only species of *Giardia* infecting humans and is comprised of eight assemblages (A-H). Among them, assemblages A and B have a broad host range and zoonotic potential. In particular, subtypes A1, A2, A3, A4, B1, and B4 are closely associated with human infections. In contrast, assemblages C-H have strong host specificity and a narrow host range [1, 8, 9]. Approximately 90% of human microsporidiosis cases are caused by *E. bieneusi* [10, 11]. In addition to its detection in humans, *E. bieneusi* has been reported in various economic animals and wildlife, including snakes and birds [12–14]. Currently, over 240 genotypes of *E. bieneusi* have been identified and divided into eight groups (groups 1–8). Most genotypes in group 1 have zoonotic potential, whereas the other groups have narrow host range and higher host specificity [15].

As an endangered species, musk deer (*Moschus* spp*.*) is currently considered a class I-protected animal in China. Forest musk deer (*Moschus berezovskii*) is the largest species of musk deer and mainly found in the Sichuan and Guizhou provinces of China [16, 17]. Musk, which has a remarkably high economic and medicinal value, is secreted by the musk gland located in the groin of male forest musk deer [18]. Because of their pathological effects on forest musk deer, infection with *G. duodenalis* or *E. bieneusi* can result in a significant loss of musk yield. This study aimed to investigate the presence of these parasites in musk deer, which may pose a threat to the health of both forest musk deer and humans.

## Methods

### Fecal sample collection

In February 2017, 223 faecal samples were collected from forest musk deer at the Sichuan Institute of Musk Deer Breeding located in Dujiangyan and Maerkang, in the Sichuan Province of China. Immediately after defecation, fresh faecal samples were collected using sterile disposable latex gloves, numbered, and placed in individual plastic bags. During specimen collection, we only gathered the top layer of the faeces to ensure that there was no contamination. All samples were placed on ice in separate containers and immediately transported to the laboratory. Specimens were stored in 2.5% potassium dichromate at 4 °C in a refrigerator until analysis.

### DNA extraction and nested PCR amplification

Faecal samples were washed with distilled water and centrifuged at 3000× *g* for 3 min. This process was repeated three times. Genomic DNA was then extracted from approximately 200 mg of each semi-purified product, using the E.Z.N.A. Stool DNA Kit (D4015–02; Omega Bio-Tek, Norcross, GA, USA). DNA samples were stored in 200 μl of the kit Solution Buffer at -20 °C until use.

*G. duodenalis* and *E. bieneusi* were identified using nested PCR amplification of the β-giardin (*bg*) gene and internal transcribed spacer (ITS) sequence, respectively. *bg*-positive specimens were subjected to further amplification of the glutamate dehydrogenase (*gdh*) and triosephosphate isomerase (*tpi*) genes, whereas ITS-positive specimens were subjected to amplification of three microsatellites (MS1, MS3, and MS7) and one minisatellite (MS4). The primers and amplification conditions were as previously described [19–21] (Table 1). Each reaction was performed in a total volume of 25 μl that included 12.5 μl 2× *Taq* PCR Master Mix (KT201-02; Tiangen, Beijing, China), 8.5 μl deionized water (Tiangen), 2 μl DNA, and 1 μl each of upstream and downstream primers. Positive and negative controls were included in each test. Secondary PCR products were subjected to 1% agarose gel electrophoresis.

**Table 1:** Primers and annealing temperature for the identification of *G. duodenalis* and *E. bieuensi*

Gene locus	Primer sequences (5’–3’)	Annealing temperature (°C)	Expected product size (bp)	Reference
*bg*	F1: AAGCCCGACGACCTCACCCGCAGTGC	50	511	[19]
	R1: GAGGCCGCCCTGGATCTTCGAGACGAC			
	F2: GAACGAACGAGATCGAGGTCCG	60		
	R2: CTCGACGAGCTTCGTGTT			
*gdh*	F1: TTCCGTRTYCAGTACAACTC	50	530	[19]
	R1: ACCTCGTTCTGRGTGGCGCA			
	F2: ATGACYGAGCTYCAGAGGCACGT	65		
	R2: GTGGCGCARGGCATGATGCA			
*tpi*	F1: AAATIATGCCTGCTCGTCG	55	530	[19]
	R1: CAAACCTTITCCGCAAACC			
	F2: CCCTTCATCGGIGGTAACTT	55		
	R2: GTGGCCACCACICCCGTGCC			
ITS	F1: GATGGTCATAGGGATGAAGAGCTT	55	392	[20]
	R1: AATACAGGATCACTTGGATCCGT			
	F2: AGGGATGAAGAGCTTCGGCTCTG	55		
	R2: AATATCCCTAATACAGGATCACT			
MS1	F1: CAAGTTGCAAGTTCAGTGTTTGAA	58	676	[21]
	R1: GATGAATATGCATCCATTGATGTT			
	F2: TTGTAAATCGACCAAATGTGCTAT	58		
	R2: GGACATAAACCACTAATTAATGTAAC			
MS3	F1: CAAGCACTGTGGTTACTGTT	55	537	[21]
	R1: AAGTTA GGGCATTTAATAAAATTA			
	F2: GTTCAAGTAATTGATACCAGTCT	55		
	R2: CTCATTGAATCTAAATGTGTATAA			
MS4	F1: GCATATCGTCTCATAGGAACA	55	885	[21]
	R1: GTTCATGGTTATTAATTCCAGAA			
	F2: CGAAGTGTACTACATGTCTCT	55		
	R2: GGACTTTAATAAGTTACCTATAGT			
MS7	F1: GTTGATCGTCCAGATGGAATT	55	471	[21]
	R1: GACTATCAGTATTACTGATTATAT			
	F2: CAATAGTAAAGGAAGATGGTCA	55		
	R2: CGTCGCTTTGTTTCATAATCTT			

### Nucleotide sequencing and analysis

Products of the expected size were sent for a two-directional sequencing analysis (Invitrogen, Shanghai, China). Assemblages and subtypes were determined by the alignment of the nucleotide sequences with known reference sequences for the *bg*, *tpi*, and *gdh* genes of *G. duodenalis*, and for the ITS, MS1, MS3, MS4, and MS7 sequences of *E. bieneusi* available in the GenBank database, using BLAST and Clustal X.

Neighbor-joining phylogenetic analysis of the aligned *G. duodenali*s and *E. bieneusi* sequences was utilised to assess genetic clustering of the genotypes. A total of 1000 replicates were used for the bootstrap analysis.

### Nucleotide sequence GenBank accession numbers

All nucleotide sequences of the *bg*, *gdh*, and *tpi* genes of *G. duodenali*s, and ITS, MS1, MS3, MS4, and MS7 of *E. bieneusi* isolated from forest musk deer in this study were deposited in the GenBank database under accession numbers MF497406–MF497412 and MF942581–MF942596, respectively.

## Results and discussion

*G. duodenalis* and *E. bieneusi* are emerging zoonotic pathogens. To our knowledge, this study is the first to report the presence of *G. duodenalis* and *E. bieneusi* in musk deer, with an infection rate of 2.24% (5/223) and 17.04% (38/223)*,* respectively. *G. duodenalis* infection rate in the Dujiangyan breeding centre (3.75%) was slightly higher than that in the Maerkang breeding centre (1.40%), whereas *E. bieneusi* infection rate was much lower in Dujiangyan than in Maerkang (7.5% and 22.38%, respectively) (Table 2). This may be due to differences in the source of food and water used for feeding, or other environmental factors.

**Table 2:** Prevalence and distribution of *G. duodenalis* and *E. bieneusi* by location in Sichuan Province, China

Pathogen	Location (city)	No. of samples	No. positive (%)	Genotype (*n*)
*G. duodenalis*	Dujiangyan	80	3 (3.75)	assemblage E
	Maerkang	143	2 (1.40)	assemblage A
	Total	223	5 (2.24)	assemblage A (2); assemblage E (3)
*E. bieneusi*	Dujiangyan	80	6 (7.50)	SC03
	Maerkang	143	32 (22.37)	SC03
	Total	223	38 (17.04)	SC03

In this study, the infected forest musk deer ranged from less than one to eight years of age. Young individuals (≤ one-year old) accounted for more than half of the *G. duodenali*s- and *E. bieneusi*-positive samples (60% and 57.89%, respectively), which may be caused by incomplete development of the immune system of young animals compared with adult animals. The proportion of infected females and males was similar. Several infected animals had obvious diarrhoea (two and nine for *G. duodenalis* and *E. bieneusi*, respectively), which may be due to the individual’s low resistance to infection. There was no apparent age- or gender-associated difference for the infections in this study, in agreement with the findings of Zhang et al. [22]. Here, assemblage A of *G. duodenalis* was obtained only from young forest musk deer in the Maerkang breeding location, whereas assemblage E was obtained from adult forest musk deer in the Sichuan Institute of Musk Deer Breeding in Dujiangyan (Table 3).

**Table 3:** Genotypes of *G. duodenalis* isolates from musk deer in Dujiangyan and Maerkang in Sichuan Province, China at the *bg*, *gdh* and *tpi* loci

Isolate	*bg*	*gdh*	*tpi*
DL12	E	E	Neg
DL26	E	Neg	Neg
DL29	E	Neg	Neg
ML55	A	Neg	A
ML117	A	Neg	Neg

*Abbreviation*: *Neg* negative

Although the distribution of *G. duodenalis* in musk deer has not been reported, there are few reports of these parasites infecting other species in the families Cervidae and Bovidae, in the same suborder as the forest musk deer. *G. duodenalis* identified in these animals was mainly assemblage A, and in several studies, the rate of infection in these species was higher than that in forest musk deer in our study. For example, Lalle et al. [23] reported that the prevalence of *G. duodenalis* was 11.5% in fallow deer (*Dama dama*) which was also higher in fawns than in older deer, and the genotype was assemblage A. García-Presedo et al. [24] reported that 8.9% of roe deer (*Capreolus capreolus*) samples were positive for *G. duodenalis*, and the genotype was AII. In Norway, 12.3% of moose (*Alces alces*), 1.7% of red deer (*Cervus elaphus*), 15.5% of roe deer, and 7.1% of reindeer (*Rangifer tarandus*) were found to be infected with *G. duodenalis* [25]. In the United States, one white-tailed deer (*Odocoileus virginianus*) was found positive for *G. duodenalis,* and assemblage A was identified [26]. Solarczyk et al. [27] reported that the sub-assemblage of *G. duodenalis* found in red deer and roe deer was AIII and zoonotic AI, respectively. Also, sheep faecal specimens from China were found to be positive for *G. duodenalis* assemblage A genotype [28]. Given that *G. duodenalis* assemblage A was previously identified in humans, forest musk deer can play a role in transmitting *G. duodenalis* to humans.

**Fig. 1:**
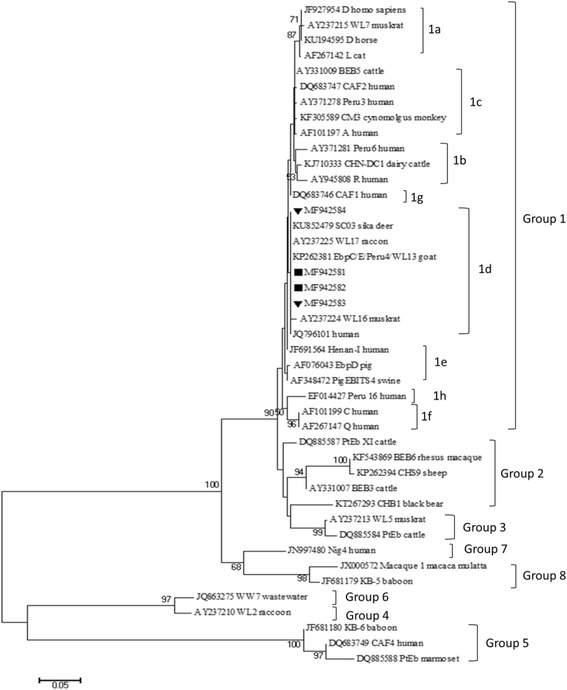
Phylogenetic relationships of the ITS gene nucleotide sequences of the *E. bieneusi* genotypes identified in this study and other reported genotypes. The genotypes in this study are indicated by triangles for Maerkang and squares for Dujiangyan

In the *E. bieneusi* analysis, ITS sequencing showed that all *E. bieneusi* isolates from Maerkang and Dujiangyan were characterised as SC03 (*n* = 38), which had been previously found in sika deer (*Cervus nippon*) at zoological gardens in China [29]. Other reference sequences in the same phylogenetic branch were from parasites isolated from racoons in eastern Maryland in the United States, goats in China, and patients with HIV/AIDS in the Henan Province of China [20, 30]. Based on the phylogenetic analysis of the ITS sequence, *E. bieneusi* isolated in this study belonged to group 1 (subgroup 1d) (Fig. 1), which suggested their zoonotic potential.

From the 38 ITS-positive specimens, there were nine, five, one, and three isolates successfully sequenced at MS1, MS3, MS4, and MS7 loci, respectively. Analysis of sequence polymorphisms and single nucleotide polymorphisms (SNPs) at MS3 locus revealed two distinct types (type I and II) (Table 4). Zhang et al. [31] reported that 7.06% (23/326) of sika deer were positive for *E. bieneusi* with eight genotypes detected. Also, 34.0% (16/47) of faecal samples from Père David’s deer (*Elaphurus davidianus*) in China were *E. bieneusi*-positive [32]. Another report showed that 29 deer were infected with *E. bieneusi*, including 28 sika deer and one red deer [33]. Zhao et al. [34] reported seven genotypes of *E. bieneusi* in golden takins (*Budorcas taxicolor bedfordi*) in China, and Shi et al. [20] found *E. bieneusi* in 28.8% (176/611) of goats and 42.8% (177/414) of sheep, with 42 genotypes identified. Twenty-three (7.0%) yaks in China were *E. bieneusi*-positive; three genotypes (BEB4, I, and J) from group 2 that were previously reported in humans and two group 1 genotypes were identified [11]. Seventeen *E. bieneusi* genotypes were identified in 26 (32.5%) white-tailed deer in the United States [26]. Therefore, the *E. bieneusi* genotype we identified in forest musk deer, and most *E. bieneusi* genotypes reported in the Cervidae and Bovidae can infect both humans and animals. However, *E. bieneusi* isolated from forest musk deer appeared to be from a single genotype, in contrast to those found in other deer species, yaks, and goats.

**Table 4:** Multi-locus sequence typing of *E. bieneusi* in musk deer

Code	Multi-locus sequence genotype					
	ITS	MS1	MS3	MS4	MS7	MLGs
LD1	SC03	ns	ns	ns	Type I	MLG1
LD48	SC03	Type I	ns	ns	ns	MLG2
LD49	SC03	ns	ns	Type I	ns	MLG3
LM50	SC03	Type I	ns	ns	Type I	MLG4
LM61	SC03	Type I	Type I	ns	ns	MLG5
LM81	SC03	ns	Type I	ns	ns	MLG6
LM83	SC03	ns	Type I	ns	ns	MLG6
LM97	SC03	ns	Type I	ns	ns	MLG6
LM102	SC03	Type I	ns	ns	ns	MLG7
LM103	SC03	Type I	ns	ns	ns	MLG7
LM104	SC03	Type I	ns	ns	ns	MLG7
LM105	SC03	Type I	ns	ns	ns	MLG7
LM123	SC03	Type I	Type II	ns	Type I	MLG8
LM131	SC03	Type I	ns	ns	ns	MLG7
Others	SC03	ns	ns	ns	ns	MLG9

*Abbreviation*: *ns* not successfully sequenced or unsuccessful, *PCR* amplification

Although the genetic heterogeneity of *G. duodenalis* and *E. bieneusi* is well described, their method of transmission is still not clear. Investigations on their epidemiology, detection methods, and diagnosis are required to provide experimental bases for ensuring the health and safety of both animals and humans.

## Conclusions

This study demonstrated the prevalence of *G. duodenalis* and *E. bieneusi* in forest musk deer in China. Furthermore, to our knowledge, this is the first report of *G. duodenalis* and *E. bieneusi* infections in musk deer and thus demonstrating that the host range of these parasites is wider than previously reported. Zoonotic genotypes identified in this study showed the transmission potential of *G. duodenalis* and *E. bieneusi* from forest musk deer to humans or other animals. Currently, there is no known effective vaccine or drug to treat infection with these parasites. Hence, measures should be taken to prevent humans and animals from being infected by these parasites.

## Abbreviations

ITS: Internal transcribed spacer
MLG: Multilocus genotype
MLGs: Multilocus genotypes
MLST: Multilocus sequence typing
SNPs: Single nucleotide polymorphisms
